# Do Farmers Using Conventional and Non-Conventional Systems of Agriculture Have Different Perceptions of the Diversity of Wild Birds? Implications for Conservation

**DOI:** 10.1371/journal.pone.0156307

**Published:** 2016-05-31

**Authors:** Horasa Lima Silva-Andrade, Luciano Pires de Andrade, Lauana Souza Muniz, Wallace Rodrigues Telino-Júnior, Ulysses Paulino Albuquerque, Rachel Maria Lyra-Neves

**Affiliations:** 1 Department of Biology, Graduate Program in Ethnobiology and Nature Conservation - PPGEtno, Federal Rural University of Pernambuco, Recife, Pernambuco, Brazil; 2 Garanhuns Campus, Federal Rural University of Pernambuco, Garanhuns, Pernambuco, Brazil; 3 Graduate Program in Management of Sustainable Local Development, University of Pernambuco, Recife, Pernambuco, Brazil; 4 Department of Biology, Laboratory of Ecology and Evolution of Social-Ecological Systems, Federal Rural University of Pernambuco, Recife, Pernambuco, Brazil; University of Oxford, UNITED KINGDOM

## Abstract

Farmers’ perceptions of birds’ interactions with agricultural production systems are fundamental to species conservation efforts. In the present study, we evaluated the perceptions of birds held by farmers who engage in conventional and non-conventional agricultural production processes and the implications of potential differences in these perceptions on species conservation. To accomplish this, data were collected using questionnaires, semi-structured interviews, and other complementary sources of information gathered from 191 farmers in northeastern Brazil. Although some similarities were identified among the farmers in their perceptions and local ecological knowledge (LEK) of birds, differences existed between the conventional and non-conventional farmers in their attitudes toward, conflicts with, and usage of bird species. Compared to the conventional farmers, the non-conventional farmers could identify more bird species, possessed more favorable attitudes toward birds, and engaged in practices more beneficial to the conservation of avifauna. The perceptions that were identified were related to the type of agriculture practiced, and such perceptions may affect the conservation of bird species. Therefore, the adoption of certain agricultural practices has important implications for conservation. Our results indicate the need for investment in public policies, programs and actions that account for farmers’ knowledge and perceptions. Such investments will contribute to the development and adoption of practices supporting wild bird conservation in agricultural areas.

## Introduction

Among all types of animals, birds are particularly vulnerable to the negative impacts of anthropogenic pressures in the ecosystems in which they live, and different bird species have different responses to such pressures. The relationships between birds and the environment have been both positively and negatively affected by the intensification of agriculture, the destruction of natural habitats [1, 2, 3], and the cultural traditions, knowledge and practices [4, 5, 6] of human populations in different regions. Birds perform primary ecological functions for agro-ecosystems as dispersers of seeds, pollinators, bioindicators, natural predators and biological controllers [7, 8, 9], and they have shown variable responses to the intensification of agriculture [10, 11]. However, farmers might not perceive these functions as valuable for the management of the agricultural systems that they adopt.

Conventional farmers primarily rely on monocultures, mechanization and pesticides, and they are strongly influenced by the agricultural model of the Green Revolution. Non-conventional farmers are influenced by agro-ecological principles and prioritize diversification in their production systems, minimizing or eliminating mechanization and the use of external inputs and instead prioritizing ecological processes and natural resource conservation [12, 13, 14]. Additionally, non-conventional farmers cultivate forested production environments, including home gardens, which are considered a form of sustainable agriculture because they provide shelter and food for native species and help conserve agrobiodiversity [15].

Different types of farming, including the specific practices adopted by farmers, may profoundly influence the local ecological knowledge held by farmers and the manner in which they perceive birds. Farmers who engage in different farming practices may also have varying attitudes toward bird conservation [12] and potential conflicts of interest with birds. Such conflicts arise when the ecological requirements and behaviors of a bird species have negative implications for humans (such as when birds cause damage to crops or livestock or pose a danger to local residents) or when human activities have negative consequences for bird populations (such as when natural bird habitats are converted into lands used for agricultural or hunting practices) [16, 17, 18].

Very little scientific research exploring how farmers perceive the birds that are present in their production systems has been published to date. For the development of agro-ecological and sustainable agriculture and to facilitate bird conservation efforts in agricultural areas [19, 20, 21, 14], it is important to consider all stakeholders involved in agricultural activities.

Therefore, the present study focused on the following questions: how do farmers (conventional and non-conventional) perceive the bird fauna found in their production systems? Do differences exist between conventional and non-conventional farmers in their perceptions of bird fauna? Do non-conventional farmers demonstrate greater knowledge of birds than conventional farmers? To what extent are farmers aware of the potential effects of their farming practices (positive or negative) on the maintenance or conservation of local bird fauna?

In the context of the above questions, we investigated the perceptions of farmers using conventional and non-conventional production systems in relation to their knowledge of wild birds and their opinions on wild bird conservation efforts. We argue that non-conventional farmers, who practice social agriculture and are guided by agro-ecological principles, possess greater ecological knowledge of local bird species and that this enhanced knowledge results in perceptions that are more favorable to bird conservation.

## Materials and Methods

### Study area

**Fig 1:**
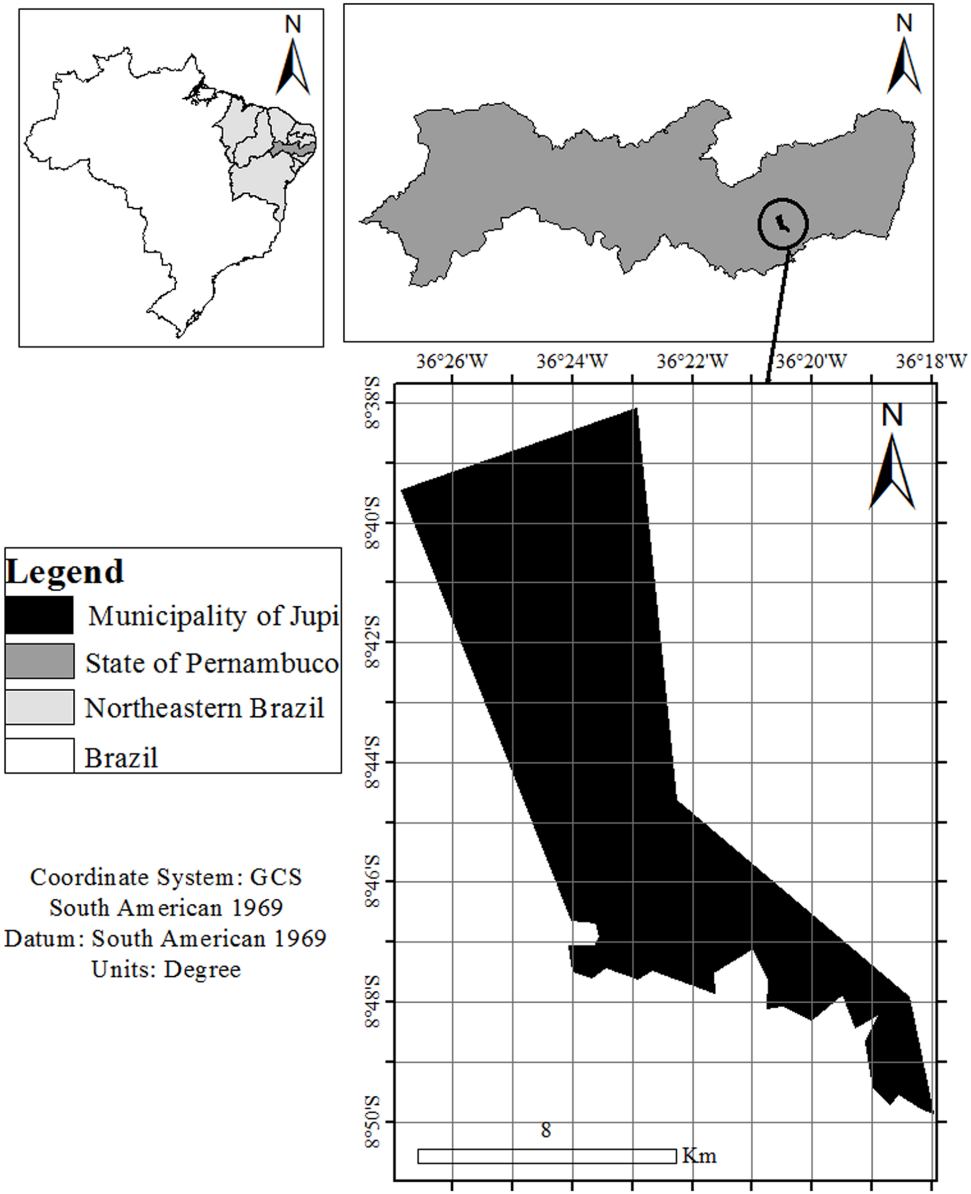
Location of the municipality of Jupi in Pernambuco, Brazil. Black—Municipality of Jupi; Dark Gray—State of Pernambuco; Light Gray—Northeastern Brazil; White—Brazil.

This study was conducted in the municipality of Jupi, Pernambuco (Fig 1), in northeastern Brazil (08°42’42” S, 36°24’54” W). The municipality has an area of 112.531 km^2^ and a population of 13,709 inhabitants [20], of which 39% reside in the rural zone and 61% reside in urban areas. The municipality is located approximately 782 m above sea level (Fig 1). The Caatinga (seasonal dry forest), consisting of deciduous and semi-deciduous forests, is the typical and predominant type of vegetation in the area, although some cloud forest formations are also present [22]. The climate is humid tropical, with a dry (austral) summer.

Public services, retail, and agriculture, including both dairy farming and the production of other crops, dominate the local economy in the study area. The rural properties in the study area vary in size from 0.5–2.0 ha to 80 ha. The majority of farms in the area are planted with conventional monocultures of beans (*Phaseolus vulgaris* L.), maize (*Zea mays* L.), and cassava (*Manihot esculenta* Crantz). However, there is an area within the municipality in which farmers maintain home gardens with abundant fruit trees (a different type of conventional agriculture), the primary role of which is to feed their families. This greener form of farming prioritizes diversification and maintains natural resources and forested environments, which in turn promote biological interactions within the agro-ecosystem [15, 23, 24].

Small regions of Caatinga habitat can still be found in Jupi, although they are under continuous pressure from the expansion of agricultural areas used for the conventional cultivation of beans, maize, and cassava. The municipality is an important producer of both beans and cassava, some of which are exported [25].

In 2009, the Brazilian government, together with local institutions, began to take action to organize and strengthen the municipality’s associations (e.g., non-profit social organizations and farmers’ collectives). This reinforced farmers’ access to the resources of the public Family Farming program and also improved agricultural productivity. These associations have contributed to the organization, participation, and empowerment of local farmers with the goal of guaranteeing the establishment of sustainable rural development.

### Data collection

The current study began in August 2013, when the details of the study were presented to local community leaders and farmers at various social and political meeting places, including collectives, clubs, syndicates, and associations. These presentations covered the objectives, methods, and procedures to be used in the study; the criteria for informant selection and participation; and the potential contributions that could be made by the farmers who choose to participate in the research. Following these meetings with local leadership, complementary criteria to define the sample design and select the informants were established.

The following study criteria were established: (i) rural areas containing both conventional farms (production based on monocultures) and non-conventional farms (e.g., household-centered agroforestry systems that may or may not have fragments of natural forest in their vicinities), and (ii) areas located within the region of the municipality that had the highest rainfall, which resulted in a higher concentration of properties with agroforestry systems.

Based on these criteria, the following associations, which are located in three different rural communities, were selected: Miné, Catonho, and Lacre. These associations were chosen because they are representative of the political organizations that farmers in the region participate in, they favor the development of the political and ethical [26] aspects of ethno-ornithological research, and they have a direct or indirect influence on improving existing strategies and developing new paradigms for species conservation [14].

A probabilistic sampling approach [27] with random selection of households [28] was used in this study. This sampling procedure focused on the heads of families (one or two per household), with the primary objective of obtaining a representative sample of households while maintaining a random sample with a 5% margin of error [28]. In total, 131 households/families were selected from the 278 residences found within the study area. From these households, a total of 191 family farmers from three different associations were selected as informants.

Following their selection, the informants were educated on the objectives of the study and confirmed their participation. After showing interest in participating in the survey, the informants signed an informed consent form (ICF) agreeing to data collection. Prior to the start of data collection, the details of the research project and the ICFs were submitted online to the Brazilian Federal Ethics in Human Studies Committee (Plataforma Brasil) and the Ethics Committee of Pernambuco State University (UPE), both of which approved and authorized the research (Protocol CAAE 30734313.0.0000.5207).

The farmers were interviewed using semi-structured questionnaires designed to obtain information about their knowledge, conflicts, uses, and practices in relation to the local bird fauna. To accomplish this, the following questions were included: What bird species are the most common in the region? What are your attitudes in relation to birds, including your conflicts with, positive/negative actions toward, and usage of birds? Do birds benefit or otherwise impact agro-ecosystems, and do such ecosystems have similar effects on bird populations? What is your perception with regard to the growth or decline of bird populations and the causes underlying these processes?

During the interviews, the farmers also provided information on the characteristics and common names of the bird species in the region. This information was recorded for later comparison with information available in the current literature and used for species identification. The species that could not be reliably identified were evaluated by expert ornithologists.

To analyze the differences in the perceptions of the two groups of farmers (i.e., conventional and non-conventional) toward birds, contingency tables were created using Excel 2010. These tables were used for subsequent data analysis using the G test in the BioEstat 5.0 software package [29] to calculate 95% confidence intervals and p values. P values <0.05 were considered significant. A PERMANOVA test was used to assess differences in the number of citations of birds among the different types of farmers. SIMPER was subsequently used to identify the species making the greatest contributions to these differences. For these analyses, the statistical program PAST 2.17c was used [30]. Similarities among the species reported by the two groups of farmers were assessed using Jaccard’s qualitative index and Sørensen’s quantitative index and were based on the number of reports obtained for each species [31].

## Results

The non-conventional farmers cited more birds than the conventional farmers. Qualitatively, the bird species reported by the two groups of farmers were 71.7% similar according to Jaccard’s index, while Sørensen’s quantitative index returned a value of 74.2%. We found a significant difference (Permutation N = 9999; Total sum of squares = 41.54; Within-group sum of squares = 37.22; F = 21.03; N = 183; DF = 182; p = 0.0001) between the numbers of species reported by the two groups of farmers. This difference was reinforced by the SIMPER analysis, which highlighted the species that made the greatest contributions to the differences found (Table 1).

**Table 1:** Bird species reported by conventional farmers and non-conventional farmers from Jupi, Pernambuco, northeastern Brazil.

Taxon	English Name	CF	NCF	Contrib	Cumulat%	MCF	MNCF
Tinamidae							
*Nothura* spp.*	Tinamou	40	14	3.426	34.69	0.374	0.184
*Crypturellus* spp.*	Grassland tinamou	42	10	3.446	29.73	0.393	0.132
Podicipedidae							
*Tachybaptus dominicus*	Least grebe	3	1	0.3121	96.12	0.028	0.0132
Ardeidae							
Bubulcus ibis*	Cattle egret	34	39	3.017	57.61	0.318	0.0921
*Ardea cocoi*	Cocoi heron	0	1	0.09744	99.46	0	0.0132
Cathartidae							
*Coragyps atratus*	Black vulture	5	7	0.941	85.6	0.0467	0.0921
Accipitridae							
*Rupornis magnirostris*	Roadside hawk	12	4	1.115	84.23	0.112	0.0526
Rallidae							
*Aramides cajanea*	Gray-necked wood rail	1	0	0.07749	99.69	0.00935	0
*Gallinula galeata*	Common gallinule	3	2	0.3873	95.15	0.028	0.0263
Charadriidae							
Vanellus chilensis*	Southern lapwing	45	32	4.2	13.97	0.421	0.421
Columbidae							
*Columbina* spp.*	Ground-dove	104	52	3.219	44.18	0.972	0.684
Cuculidae							
*Coccyzus melacoryphus*	Dark-billed cuckoo	1	3	0.357	95.66	0.00935	0.0395
Crotophaga ani*	Smooth-billed ani	25	22	3.05	53.23	0.234	0.289
*Guira guira*	Guira cuckoo	3	0	0.2928	96.98	0.028	0
*Tapera naevia*	Striped cuckoo	0	1	0.08462	99.58	0	0.0132
Strigidae							
Athene cunicularia*	Burrowing owl	13	23	2.843	61.73	0.121	0.303
Caprimulgidae							
*Antrostomus rufus*	Rufous nightjar	0	1	0.115	99.32	0	0.0132
Trochilidae							
*Eupetomena macroura*	Swallow-tailed hummingbird	0	1	0.07074	100	0	0.0132
*Amazilia* spp.*	Hummingbird	18	14	2.272	72.72	0.168	0.184
Picidae							
*Veniliornis passerinus*	Little woodpecker	1	1	0.1904	98.34	0.00935	0.0132
Falconidae							
*Caracara plancus*	Southern crested caracara	6	3	0.7613	87.81	0.0561	0.0395
*Herpetotheres cachinnans*	Laughing falcon	1	0	0.07134	99.79	0.00935	0
Psittacidae							
Psittacara leucophthalmus*	White-eyed parakeet	13	27	3.324	39.51	0.121	0.355
*Eupsittula cactorum*	Caatinga parakeet	3	5	0.7184	89.96	0.028	0.0658
*Forpus xanthopterygius*	Blue-winged parrotlet	0	3	0.2598	97.77	0	0.0395
Furnariidae							
*Furnarius* spp.	Hornero	2	0	0.1436	98.55	0.0187	0
Phacellodomus rufifrons*	Rufous-fronted thornbird	20	8	2.023	75.65	0.187	0.105
Tyrannidae							
*Pitangus sulphuratus*	Great kiskadee	16	25	1.795	78.25	0.15	0.118
Fluvicola nengeta*	Masked water tyrant	36	27	3.868	19.58	0.336	0.355
Turdidae							
*Turdus* spp.	True thrush	7	11	1.365	82.62	0.0654	0.145
Motacillidae							
*Anthus lutescens*	Yellowish pipit	3	1	0.3036	96.56	0.028	0.0132
Passerellidae							
*Zonotrichia capensis*	Rufous-colored sparrow	4	4	0.5992	90.82	0.0374	0.0526
Icteridae							
*Icterus pyrrhopterus*	Variable oriole	0	1	0.07074	99.9	0	0.0132
*Gnorimopsar chopi*	Chopi blackbird	0	1	0.07074	100	0	0.0132
*Molothrus bonariensis*	Shiny cowbird	2	2	0.2876	97.4	0.0187	0.0263
*Sturnella superciliaris*	White-browed blackbird	1	0	0.5107	93.95	0.028	0.0395
Thraupidae							
*Coereba flaveola*	Bananaquit	0	8	0.7666	86.71	0	0.105
*Lanio pileatus*	Pileated finch	2	0	0.1318	99.15	0.0187	0
*Tangara cyanocephala*	Red-necked tanager	1	1	0.1397	98.96	0.00935	0.0132
*Tangara* spp.	Tanager	3	3	0.5502	92.46	0.028	0.0395
Paroaria dominicana*	Red-cowled cardinal	20	16	2.48	69.43	0.187	0.211
*Sicalis flaveola*	Saffron finch	10	10	1.647	80.64	0.0935	0.132
*Sicalis luteola*	Grassland yellow finch	7	1	0.5765	91.66	0.0654	0.0132
*Volatinia jacarina*	Blue-black grassquit	7	3	0.7605	88.91	0.0654	0.0395
Sporophila nigricolis*	Yellow-bellied seedeater	30	27	3.558	24.73	0.28	0.355
Sporophila albogularis*	White-throated seedeater	24	18	2.831	65.83	0.224	0.237
Sporophila leucoptera*	White-bellied seedeater	6	29	3.2	48.81	0.0561	0.382
*Sporophila brouvreuil*	Copper seedeater	5	2	0.5159	93.21	0.0467	0.0263
Cardinalidae							
*Piranga flava*	Hepatic tanager	1	1	0.1411	98.76	0.00935	0.0132
*Cyanoloxia brissonii*	Ultramarine grosbeak	2	3	0.4418	94.59	0.0187	0.0395
Fringillidae							
*Sporagra yarrellii*	Yellow-faced siskin	1	2	0.2011	98.07	0.00935	0.0263
*Euphonia chlorotica*	Purple-throated euphonia	0	2	0	100	0	0
Passeridae							
Passer domesticus*	House sparrow	43	76	5.439	7.883	0.402	1
Total number of species and reports		44/663	47/577				

CF, conventional farmers; NCF, non-conventional farmers; SIMPER: Contrib, contribution; Cumulat, cumulative %; MCF, mean conventional farmers; MNCF, mean non-conventional farmers.

*Species that were reported considerably more often by one group of farmers relative to the other.

The non-conventional farmers were more knowledgeable about the birds found on their properties than the conventional farmers and presented significantly more positive attitudes toward potential situations of conflict (G = 13.5507; DF = 3; p = 0.0036; Table 2). Evidence of these positive attitudes included not authorizing hunting and allowing birds to remain free due to the beauty of their plumage or song.

**Table 2:** Analysis of the questions presented to the conventional and non-conventional farmers to assess their attitudes, conflicts, uses, perceptions of benefits/harm and perceptions of increases/reductions in numbers with regard to local bird species.

QA/SA (G test)	CF	NCF	Specific answers	
			CF	NCF
Question 1/G = 13.5507; DF = 3; p = 0.0036	Negative: 3	Negative: 24	Sparrows are harmful: 0	Sparrows are harmful: 3
			Kill: 1	Kill: 6
			Hunt: 20	Hunt: 10
			Sell: 0	Sell: 1
			Pets: 7	Pets: 0
			Use pesticides: 1	Use pesticides: 3
			Kill/Sell: 2	Kill/Sell: 1
	Positive: 19	Positive: 28	Protect/Conserve: 16	Protect/Conserve: 21
			Enjoy their presence: 1	Enjoy their presence: 3
			Leave free: 2	Leave free: 4
	No Attitude: 30	No Attitude: 39		
	Don’t know/No opinion: 11	Don’t know/No opinion: 1		
Question 2/Yes/No, G = 5.3986; DF = 1; p = 0.0202; Types of conflict G = 17.4168; DF = 3; p = 0.0006	Yes: 4	Yes: 16	Pets: 9	Pets: 1
			Hunting for food, sport or illegal trade: 17	Hunting for food, sport or illegal trade: 0
			Sparrow: 1	Sparrow: 2
			Kill: 0	Kill: 2
	No: 61	No: 70		
	No opinion: 2	No opinion: 1		
Question 3/G = 24.5598; DF = 4; p < 0.0001			Pets: 13	Pets: 2
			Food: 28	Food: 11
			Illegal trade: 3	Illegal trade: 10
	None: 44	None: 64		
	Don’t know/No opinion: 3	Don’t know/No opinion: 2		
Question 4/Increase/Reduction G = 0.5287; DF = 3; p = 0.9125; Causes a reduction G = 53.3775; DF = 11; p < 0.0001; Causes an increase G = 12.2811; DF = 4; p = 0.0154	Reduction: 14	Reduction: 13	Deforestation: 9	Deforestation: 25
			Hunting: 9	Hunting: 15
			Drought: 24	Drought: 19
			Human actions: 2	Human actions: 1
			Use of pesticides: 3	Use of pesticides: 9
			Predation: 1	Predation: 4
			Migration: 0	Migration: 1
			Drought/Deforestation: 13	Drought/Deforestation: 0
			Drought/Use of pesticides: 0	Drought/Use of pesticides: 5
			Hunting/Use of pesticides: 0	Hunting/Use of pesticides: 2
			No answer: 0	No answer: 2
			None: 0	None: 9
	Increase: 19	Increase: 18	Do not hunt anymore: 7	Do not hunt anymore: 7
			Conservation: 1	Conservation: 5
			Planting trees: 1	Planting trees: 1
			No answer: 3	No answer: 0
			None: 3	None: 0
	No effect: 33	No effect: 28		
	Don’t know/No opinion: 31	Don’t know/No opinion: 34		
Question 5/ G = 7.5147; DF = 3; p < 0.0572	Benefit: 21	Benefit: 27		
	Harm: 10	Harm: 5		
	None: 46	None: 49		
	Don’t know/No opinion: 20	Don’t know/No opinion: 8		
Question 6/ G = 8.5146; DF = 3; p < 0.0365	Benefit: 39	Benefit: 39		
	Harm: 9	Harm: 8		
	None: 41	None: 45		
	Don’t know/No opinion: 6	Don’t know/No opinion: 0		

QA, Questions analyzed; SA, Statistical analysis; CF, conventional farmers; NCF, non-conventional farmers; Question 1, Attitudes toward bird fauna; Question 2, Conflicts; Question 3, Uses; Question 4, Increases/reductions in bird populations according to the type of farming practiced; Question 5, Benefits/harm of the farming system to the birds; Question 6, Benefits/harm of the birds to the farming system.

With regard to the conflicts that existed between farmers and the local bird fauna, the non-conventional farmers made fewer references to conflicts, although they reported a significantly higher number of conflicts than the conventional farmers (G = 5.3986; DF = 1; p = 0.0202; Table 2). The reported conflicts included problems provoked by the behavior of the birds (affecting the objectives of the farmers) and the negative impacts of the farmers’ interests in and objectives regarding the birds (G = 17.4168; DF = 3; p = 0.0006; Table 2).

The few conflicts mentioned by both groups of farmers may be characterized as the result of negative anthropogenic impacts on the birds. For example, the conflicts included the capture of species such as the white-throated seedeater, yellow-bellied seedeater, and saffron finch, which were kept as pets, as well as the hunting of birds for food, such as ground-doves, grassland tinamous and tinamous. The farmers also referred to the effects of the birds on productivity; for example, species such as house sparrows and parakeets were known to attack crops, while species such as caracaras were known to prey on livestock. Sparrows also invaded houses, and the threat of disease transmission resulted in the killing of many birds.

The non-conventional farmers reported significantly less usage of birds than the conventional farmers (G = 24.5598; DF = 4; p < 0.0001; Table 2). The conventional farmers referred to subsistence hunting more frequently than the non-conventional farmers (Table 2); they reported using birds for food and as pets, as well as illegally trading certain species.

In general, neither group of farmers noted a perception of an increase or reduction in bird populations; in fact, most informants answered “don’t know” or “no opinion” when questioned about this issue (Increase/Reduction G = 0.5287; DF = 3; p = 0.9125; Table 2). The conventional farmers perceived a growth or decline in the abundance of birds slightly more frequently than the non-conventional farmers, although the difference was not statistically significant. However, when the informants were asked to identify the causes of a perceived increase (G = 12.2811; DF = 4; p = 0.0154; Table 2) or decrease (G = 53.3775; DF = 11; p < 0.0001) in bird populations, the difference between the two groups was highly significant. Compared to the conventional farmers, the non-conventional farmers stated much more frequently that reductions in bird populations were due to negative anthropogenic impacts (such as deforestation) as well as direct actions (such as hunting and the use of pesticides). They also cited climatic factors such as drought as well as the behavioral characteristics of the birds themselves, particularly with respect to migration. The factors identified as responsible for causing an increase in the abundance of species included conservation initiatives on the part of the farmers, such as the protection of nests found within their production systems.

Both groups of farmers cited reduced hunting and increased tree planting as activities that contribute to an increase in bird populations. More non-conventional than conventional farmers mentioned that positive attitudes regarding the conservation of species contribute to an increase in local bird populations.

With regard to the relationships that exist between birds and agro-ecosystems (G = 7.5147; DF = 3; p < 0.0572; Table 2), the non-conventional farmers reported more often than the conventional farmers that their systems of production bring more benefit than harm to different bird species. The non-conventional farmers made more references to the benefits their farming systems could provide to local birds, rather than the harm their systems could cause. A much larger number of conventional farmers had either no opinion or no knowledge in response to this question.

When asked about the potential benefits/harms associated with local birds with respect to the different agricultural systems practiced (G = 8.5146; DF = 3; p < 0.0365; Table 2), similar numbers of the conventional and non-conventional farmers referred to the benefits provided by the birds, although more of the conventional farmers had no opinion on the question or responded that the birds harmed their agro-ecosystems. In their responses to questions regarding their relationships to the bird fauna (e.g., attitudes, conflicts, and uses) and their perceptions of the interactions that exist between birds and their production systems (e.g., increases/reductions in species related to the type of farming practices and benefits/damage to birds caused by these practices and vice versa), a large number of the interviewees reported that no systematic relationship exists (“none”).

The conventional and non-conventional farmers’ perceptions of birds clearly differed (Table 2), and these differences were often highly statistically significant. Indeed, a non-significant difference was only noted with regard to the farmers’ perceptions on whether an increase or decrease in species number was the result of a specific type of farming practice.

## Discussion

This study demonstrated that conventional and non-conventional farmers possess different perceptions of regional bird fauna. While both groups of farmers recognized the birds present in their corresponding agro-ecosystems, the non-conventional farmers recognized a slightly larger number of species than the conventional farmers. Jacobson et al. [12] recorded similar findings when comparing organic and conventional farmers in Florida, where the former recognized a larger number of birds, presumably because a larger number of species were found in their production systems. These authors also concluded that both conventional and organic farmers regarded their properties as good habitats for birds, particularly for the conservation of species that have suffered negative impacts from the intensification of farming activities in the region.

While the perceptions of the conventional and non-conventional farmers in this study with regard to the birds present in their production systems were 70% similar, there were significant differences at the level of species recognition. The birds most often cited by the conventional farmers were those most targeted by the local population as sources of food (e.g., ground-doves, tinamous, and grassland tinamous) and/or pets (e.g., white-throated seedeater and rufous-fronted thornbird), those found in their production systems (e.g., masked water tyrant, smooth-billed ani, guira cuckoo, and southern lapwing), and predatory species, such as hawks, which attack livestock. The perceptions of this group of farmers reflect a more predatory relationship with birds; such a relationship may be sustained and reinforced by socio-economic factors and cultural traditions [32, 33, 34, 35]. This characteristic may be related to the use of monoculture-based agricultural systems, which tend to attract species that are omnivorous generalists, such as tinamous, southern lapwings, and anis. This results in reduced richness of bird species, either due to the reduced diversity of plant life or because monocultures support species that exploit the natural vegetation found in these areas [36, 37, 11].

The non-conventional farmers cited more species living in the vegetation in their production systems (e.g., ground-dove, cattle egret, southern lapwing and white-bellied seedeater), as well as the invasive house sparrow. These farmers often referred to species that visit their production systems in search of resources (roosts, food, and reproductive sites) rather than those that have some functional relationship with the agricultural environment, which were more often cited by the conventional farmers.

In this case, the non-conventional farmers’ perceptions of the birds found in the vegetation in their production systems were consistent with findings reported by Freemark and Kirk [13] and Goulart et al. [37], who concluded that diverse systems, such as agroforesty orchards and plots, contain a large diversity of birds. These birds use the existing vegetation for refuge, nesting, foraging, and other activities, particularly in the case of species that are dependent or semi-dependent on forested habitats [38]. In this case, incentives for the adoption of more sustainable farming practices, including non-conventional systems, will contribute to the conservation of certain components (i.e., species) of local bird diversity in the agricultural areas [39, 10] that provide the most distinct types of landscapes.

An understanding of the relationship that exists between birds and agricultural systems may or may not contribute to the development of conservation measures, as shown by Herzon and Mikk [40], who found that farmers may develop a number of different relationships with birds, ranging from attracting them to their plantations for pest control (which also guarantees the conservation of their populations) to eliminating them as agricultural pests due to the damage they cause to crops.

In the current study, the conventional farmers tended to have a better understanding of the birds with which they had a functional relationship. Capture and exploitation of these species was more common, and the farmers also demonstrated greater practical knowledge of predatory practices such as hunting and trapping of birds for personal pets or illegal trade. This was confirmed during the interviews, when many of the informants described being caught hunting or keeping birds in captivity. Alves et al. [32] and Williams et al. [35] affirm that the knowledge possessed by local human populations regarding the behavior of certain bird species is one factor that may contribute to the continuation of predatory activities, such as hunting, that result in declining populations of target species. It is thus important to consider the implications of such activity when planning conservation strategies.

In the present study, the most commonly cited conflicts were related to the pursuit and hunting of bird species for a number of different uses by the farmers. Other conflicts related to the extermination of certain species. One such example is the house sparrow, which causes damage to crops and carries the risk of disease transmission. Sparrows are killed using firearms and poison or white rats as a form of biological control. Sick [36] characterized the house sparrow as a highly aggressive invasive species that withstands anthropogenic modifications of the environment due to its generalist and opportunistic behavior. The invasive habits of this species have contributed decisively to its persecution by the local farmers.

Falconiformes, which attack smaller livestock animals, are also persecuted by the local population and were cited by the farmers as a source of conflict. A similar scenario has been discussed by Alves et al. [4], Fernandes-Ferreira [34], and Mendonça et al. [18]. Conflicts arise between farmers and birds as a result of birds attacking crops, leading to negative attitudes toward some species, as reported by Herzon and Mikk [40] and Alves et al. [4]. Weladji and Tchamba [16] have described attacks on crops by a species of true parrot (Psittacidae) in Cameroon, Africa. In this case, the local farmers cited deforestation as a major cause of the disappearance of the region’s birds, although this reduction of habitat was not recognized by the farmers as a conflict. While the destruction of the habitat of burrowing owls is not mentioned in the present study, we did observe this activity and classified it as a conflict: the interests of the farmers and their need to plant crops conflicts with preservation of the owl’s habitat. Such a perspective should be considered in any conservation plan.

Sparrows have become pests in the region studied, and the farmers there believe they may cause harm to human health. However, despite these perceptions, the birds do not cause injury to humans. Their populations have sharply increased and, because of their behavioral characteristics [36], they can cause environmental disturbances by crowding out other native species.

Both groups of farmers were reluctant to explicitly discuss the predatory practices that still occur in the region, probably due to their knowledge of federal legislation (Environmental Crimes, Brazilian federal law 9.605/1998) [41] prohibiting the hunting, trapping or commercial exploitation of wild birds. The Chico Mendes Institute for the Conservation of Biodiversity, or ICMBio [42], recognizes hunting as a major direct threat to the birds of the Caatinga (the biome in which Jupi is located) and reinforces the need for the development of conservation and environmental education programs, along with other action plans for species conservation.

The farmers’ perceptions of bird species resulted from the types of farming methods they adopted. As reported by other authors, non-conventional farmers have developed agricultural practices more favorable to avifauna and biodiversity conservation [15]. Their farming systems prioritize complexity, ecological interactions and effective use of natural resources. In addition, the diversification of their production systems contributes to the richness and maintenance of bird species [43, 44, 23].

Adopting a particular type of agriculture affects whether avifauna biodiversity is maintained and therefore has conservation implications. Thus, certain types of agriculture, along with the practices, knowledge, and attitudes of the corresponding farmers in relation to birds, are crucial to the preservation or destruction of birds in agricultural areas.

Non-conventional agriculture and the knowledge and practices resulting from it are more favorable to the conservation of local birds [13]. This type of agriculture supports the development of social farming and the sustainable maintenance of biodiversity [23]. It requires specific practices and strategies and the implementation of public policies and processes that promote the conservation of bird species in agricultural areas [8, 39, 21, 40].

In this context, Nolan and Robbins [45], Toledo and Barrera-Bassols [46], Shen et al. [47], and Kai et al. [48] have emphasized that orally transmitted cultural traditions and the lifestyles of traditional farming populations may influence local ecological knowledge, interactions, and positive or negative actions toward species. This may or may not contribute to species conservation. The perceptions and attitudes of farmers in relation to the wild birds found in their production systems are determined by the farming practices and types of production systems adopted. These perceptions may be influenced by both cognitive and emotional factors, including cultural traditions [45, 46].

Toledo and Barrera-Bassols [46] concluded that the perceptions of traditional populations are molded by a combination of beliefs, knowledge, and practices. These combine to form local ecological knowledge, which is transmitted orally and shifts constantly from one generation to the next due to cultural and temporal changes. This process needs to be better understood and investigated through ethno-ornithological research, which will support the development of more effective strategies for the conservation and management of biodiversity.

Given the above issues, it is necessary to analyze the knowledge and practices of traditional farmers who have adopted agricultural practices that prioritize either the simplification or the diversification of their systems, as well as the implications of these systems for the wild birds that occupy the corresponding farms. Depending on the type of farming adopted and the agricultural practices of local farmers, local ecological knowledge may reinforce cultural traditions, attitudes, and practices that contribute to the increase/reduction, maintenance/decimation or conservation/extinction of local bird species. In southwestern China, Shen et al. [47] concluded that the traditional practices and local ecological knowledge of the population must be taken into consideration when developing conservation strategies given that these practices, more so than the knowledge transmitted by formal education, are favorable to the conservation of biodiversity and the protection of bird populations. In Jupi, non-conventional farmers follow traditional practices that are more favorable to the conservation of bird populations, as observed by Shen et al. [47]. The practices and traditions of the local populations examined in both the current study and that reported by Kai et al. [48] can be used to develop educational programs and government actions that stimulate the members of the community to explore their cultural memories, thus putting local ecological knowledge to use in conservation.

The non-conventional farmers had more positive attitudes with regard to bird conservation: they explained that they enjoyed watching birds and preferred to allow them to go free, recognizing the beauty of their songs and plumage. These individuals frequently used the word preservation when referring to the conservation of species and commented on the human actions that negatively impact the environment and birds in general. This shows a greater sensitivity toward environmental questions and indicates enhanced awareness of environmental changes occurring as a consequence of human activities. This same group mentioned that social programs and public policies implemented by the government contribute to a reduction in predatory practices such as hunting and trapping. The local populations receive financial assistance, which has resulted in an improvement in their quality of life and a reduction in the need to hunt and trap birds. In addition, television programs, educational campaigns, and other minor efforts all contribute to species conservation. For example, Nekaris et al. [49] and Kai et al. [48] found that both the media and environmental education programs may influence perceptions and knowledge of local fauna, which can then be used to support the conservation of regional biodiversity.

In our research, the most frequently cited environmental conflicts were the persecution and hunting of bird species by farmers and the slaughter of species (e.g., the sparrow) that cause damage to crops and carry the risk of transmitting disease to humans. It is worth noting that Falconiformes, although not frequently cited by our informants, do warrant attention because they are persecuted and killed by the local population.

The farmers in the present study were not aware of the potential interactions (beneficial or damaging) that existed between their production systems and the local bird fauna. This is consistent with the results of Jacobson et al. [12], who found that 91% of their interviewees did not see the potential for using birds to provide biological control on their plantations. In Jupi, only a few farmers, principally those practicing non-conventional agriculture, reported benefits or damage to their agro-ecosystems caused by birds (or vice versa). This emphasizes the need to develop strategies and learning environments that explore the biological interactions between bird fauna and agro-ecosystems, as well as the need to adopt appropriate measures of conservation and management for local bird populations. Herzon and Mikk [40] and Jacobson et al. [12] found that most farmers are willing to adopt practices that attract birds and contribute to species conservation in the context of their production systems.

In the current study, the relationships that were established by the farmers between their production systems and the local bird populations were limited to the biological control of insects by egrets, sparrows, and anis and the impact of deforestation on agricultural plots. Both of these interactions can support either the growth or reduction of bird populations, depending on the species. Although the non-conventional farmers were expected to identify more beneficial relationships between the birds and their production systems (and vice versa), this was not confirmed by the results of the study. Instead, the study showed that while these farmers do recognize a larger number of species and have greater knowledge of and attitudes more favorable to bird conservation in rural areas, they do not explicitly comprehend the benefits of either their agro-ecosystems or more traditional farming practices for the diversification of bird habitats or the implications of this habitat diversification for the conservation of local bird diversity. Therefore, it is important to develop integrated conservation and education programs in collaboration with local farmers to ensure the conservation of wild bird populations and to stimulate sustainable farming practices. This should include the recognition, preservation, and valorization of the local ecological knowledge of traditional populations [46]. In this context, Wood et al. [50] concluded that innovative and transformative agricultural practices can be developed through the assimilation of new knowledge through the integration of the actions of farmers, entrepreneurs, government agents, and scientists.

The conventional farmers in the current study demonstrated less interest and less specific knowledge with regard to the interactions that exist between the local bird fauna and their production systems. They identified few potential ecological functions for this group of animals in the context of local agro-ecosystems, such as bio-indication, biological control, seed dispersal, pollination, and the birds’ potential roles in restoration ecology. Jacobson et al. [12] found that farmers who adopted more sustainable and social agricultural practices recognized possible interactions between birds and their productive systems much more frequently than conventional farmers and were more willing to adopt conservation-oriented measures and practices. However, we expected that the non-conventional farmers’ recognition of the importance of the local bird fauna and their potential functions in agro-ecosystems would be stronger than it actually was.

The conventional farmers identified the principal causes of reductions in bird populations as (i) the region’s semi-arid climate, and (ii) the negative effects of deforestation. Arid climates and deforestation do, in fact, affect a region’s bird populations [38]. However, this group of farmers did not recognize the negative impacts of their attitudes and practices, such as hunting and trapping, on local bird populations. In this case, environmental education would help the farmers better understand that their attitudes and practices may have negative consequences for local bird populations. Herzon and Mikk [40] and Jacobson et al. [12] have emphasized the need to ensure that farmers understand the negative consequences of agricultural intensification on local bird populations. Such measures should help promote farmers’ interest in wildlife conservation and encourage them to adopt appropriate measures that could be stimulated by public policies, educational materials, technical assistance from government agencies, and other incentives. The development of an educational program integrating government agencies and farmers should also take into account the interactions that exist between scientific knowledge and traditional knowledge and practices [46, 50]. Traditional knowledge and practices can help contribute to the definition of new paradigms of production, supported by the principles of agroecology and ethnoecology.

The non-conventional farmers in the current study attributed the decline in local bird populations to anthropogenic pressures on the environment and to the birds themselves, reflecting a clearer and more integrated perception of how human actions affect species richness and diversity. While we found no significant difference between the conventional and non-conventional farmers in terms of their perceptions of the relationships that exist between their farming practices and increases/reductions in bird populations, their practices, attitudes toward conservation, and motivations all varied significantly. As they practice a more sustainable type of farming, the non-conventional farmers were far more knowledgeable with regard to local birds than were the conventional farmers; this knowledge favors the conservation of species [12].

In addition, the non-conventional farmers also had a better understanding of the negative consequences of human activities on bird populations, such as the declines observed in some species, as well as the increased numbers of invasive species, such as the sparrow, which withstands deforestation [36]. A similar pattern has been observed by Jacobson et al. [12], Bolwing [19], Herzon and Mikk [40], and Teillard et al. [11], reinforcing the need for further research into how farming systems and local bird fauna can be more systematically integrated.

The potential importance of birds for farming systems emphasizes the need to develop integrated measures that reinforce the recognition of the roles these animals play in both the natural ecosystem and in rural areas, as well as their potential contributions to more sustainable farming practices [12, 8, 23]. In particular, it is essential to understand that bird conservation and farming practices are correlated [12] and that bird populations must be seen as an important element of the agro-ecosystem: they perform specific ecological functions, promote positive interactions, and provide potential benefits to agricultural systems.

## Conclusions

Conventional and non-conventional farmers have both similarities and differences in their perceptions and knowledge of birds. There are incentives to help farmers recognize and treat birds as beneficial to farming systems and vice versa. Birds are still not widely recognized as important elements that could serve fundamental ecological functions in agro-ecosystems and contribute to their efficiency and sustainability. New research should prioritize evaluating the local knowledge of traditional populations to understand why farmers do not report knowledge of birds’ ecological functions and the implications of such knowledge on conservation.

Clearly, strategies for the conservation and management of regional bird fauna must be integrated with the adoption of sustainable farming practices and should be based on farmers’ perceptions of birds and their interactions with systems of agricultural production. The knowledge, practices, and attitudes of farmers must be taken into consideration to promote the conservation of bird populations in rural areas.
